# Development of therapeutic digestive endoscopy robots: a narrative review

**DOI:** 10.20452/wiitm.2024.17907

**Published:** 2024-07-12

**Authors:** Yaxian Kuai, Jing Li, Zhaoyi Chen, Bin Sun, Xu Wang, Derun Kong

**Affiliations:** Key Laboratory of Digestive Diseases of Anhui Province, Department of Gastroenterology, The First Affiliated Hospital of Anhui Medical University, Hefei, China

**Keywords:** endoscopic submucosal dissection, endoscopy, minimally invasive surgery, robotics, traction

## Abstract

**INTRODUCTION:**

With the continuous development of science and technology, robotics technology has gradually penetrated into various fields of application. In medicine, it is widely used in surgery to improve procedural accuracy and patient safety.

**AIM:**

This paper aims to explore the application of robotics in endoscopic submucosal dissection (ESD) and to summarize the principles, techniques, advantages, and limitations of robot-assisted procedures.

**MATERIALS AND METHODS:**

A comprehensive search of the PubMed database and was conducted since the database inception until March 2024. We performed the search using subject headings and key words to obtain relevant articles focused on the application of robotic systems in ESD.

**RESULTS:**

A total of 21 studies describing 9 robotic systems were found eligible. The systems were categorized into one-type endoscopic robots and integrated endoscopic robots, and individual systems were reviewed and compared in a narrative form.

**CONCLUSIONS:**

Robotics technology has essential application value and development potential in ESD. Improving the accuracy and stability of the robotic arm, reducing its cost, and broadening its applica tion are the key challenges faced by this continuously advancing technology. It is necessary to further strengthen technology research, development, innovation, and interdisciplinary cooperation to promote the use and advancement of robotics technology in ESD.

## INTRODUCTION

Endoscopic submucosal dissection (ESD) is a minimally invasive surgical method developed in Asia in the middle to late 1990s.^1^^;^^2^^;^^3^ It can be used to effectively remove large and irregular tumors, with high overall and R0 resection rates.^4^ ESD has been successfully utilized for a variety of indications, such as resecting the entire gastrointestinal tract and early invasive mucosal-based gastrointestinal tumors.^5^ Performed using a traditional endoscope, it is an innovative, yet technically demanding procedure. However, due to a lack of reverse traction exposure of the submucosal dissection plane, the operative time is prolonged, with increased risks of bleeding and perforation. This requires experience and skills from surgeons, thereby limiting the widespread adoption of ESD.^4^^;^^6^^;^^7^^;^^8^ Recently, there have been advancements in equipment and traction methods for ESD procedures,^9^^;^^10^^;^^11^^;^^12^ including position traction, heavy traction, hemostatic clamp combined with elastic circle traction, S-O metal clip traction, magnetic traction, percutaneous traction, and others.^13^^;^^14^^;^^15^^;^^16^^;^^17^^;^^18^^;^^19^^;^^20^^;^^21^^;^^22^ However, these methods, have limitations with respect to traction direction, tension, and tissue grasping, making them suitable only for specific areas. Robotassisted ESD plays a prominent role in modern medicine.

The medical field where robots were first used was surgery. Thanks to its continuous development and innovation, natural orifice transluminal endoscopic surgery (NOTES) has led to advancements in gastrointestinal disease diagnosis and treatment, making procedures less invasive.^23^ This has also contributed to the progress of digestive endoscopy robots.^24^ These robots feature examination and therapeutic systems. A majority of digestive endoscopic surgical robots are of the master-slave type. Depending on whether the endoscope-carrying platform is integrated with ordinary endoscopes or not, the robots are classified as either one-type or integrated endoscopic robots.

**FIGURE 1:**
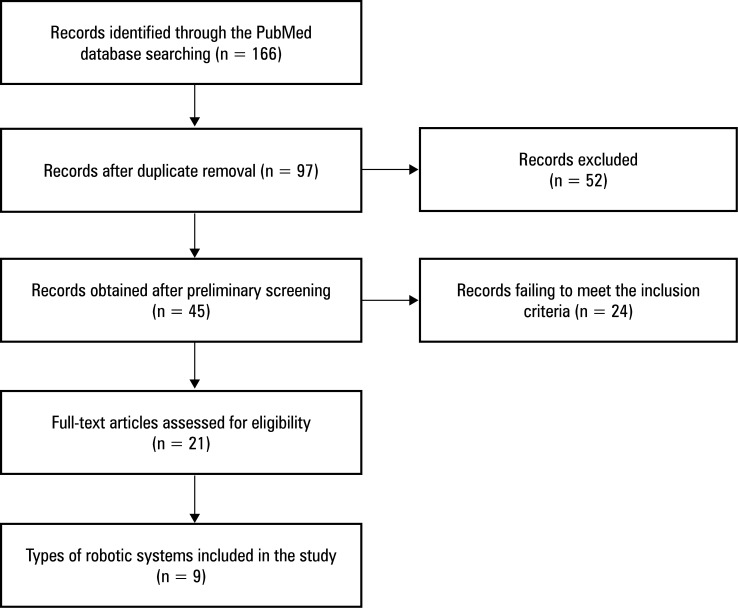
Flowchart of the search strategy

## AIM

This review focuses on the structural characteristics and clinical performance of therapeutic endoscopic robotic systems used in ESD. The aim is to offer insights into the development and clinical implementation of therapeutic digestive endoscopic robots, as well as to encourage a more widespread use of ESD technology.

## MATERIALS AND METHODS

 We conducted a comprehensive literature search of the PubMed database since its inception until March 2024, mainly focusing on studies and reports on the application of robotics in ESD. We used a search strategy combining Medical Subject Heading terms, subject terms, and key words, such as endoscopy, robotics, ESD, therapy, traction, surgery, and others to obtain relevant articles. A summary of the study selection process is shown inFIGURE 1.

### Ethics 

The study was approved by the Ethics Committee of the First Affiliated Hospital of Anhui Medical University (PJ 2023-12-12). Written informed consent from the patients was not required.

#### Statistical analysis

 A descriptive analysis was carried out. The categorical variables were expressed as numbers and percentages and the quantitative variables were expressed as mean and SD.

## RESULTS 

A total of 21 studies were found eligible for inclusion in the study. They described the application of 9 robotic systems, which were further divided into one-type endoscopic robots (n = 3) and integrated endoscopic robots (n = 6). Individual robot types were reviewed and compared in a narrative form, as shown in TABLE 1.

### One-type endoscopic robots

####  Subperichondrial transseptal system 

Subperichondrial transseptal system (STRAS) is a master-slave robotic system that was modified and developed in 2013 by French researchers, Donno and colleagues, based on the Anubiscope endoscopic platform.^24^^;^^25^^;^^26^^;^^27^^;^^28^ It offers 10 degrees of freedom (DOF) for rotation and translation, enabling 3-dimensional (3D) positioning of the endoscopic camera. However, it has certain shortcomings, such as the inability to perform complete partial rotational movements and limited accuracy in small-amplitude motions. The system was refined by ^28^ in 2017. The second generation of STRAS consists of an endoscope, 2 operating arms, a rotation / translation module, a U-shaped stent, and a moving cart.^29^ T he distal endoscope features a standard configuration, including cameras, a lighting system, and an air channel with a central channel diameter of 3.2 mm. Two flexible arms, each with a dedicated channel diameter of 4.3 mm, extend from the primary lens end on both sides, and they are capable of combining at the tip to form a closed loop. A streamlined shell protects the outer edge from damage during insertion. Three-position actuators are utilized for grasping, cutting, and wound suturing during procedures. In a live pig study, STRAS successfully performed a complete colon ESD procedure. Another live study involving 12 colon ESD cases confirmed the system’s feasibility, with an average dissection time of 34.25 minutes (range, 4–93 minutes) and a significantly higher dissection speed, as compared with traditional endoscopy (mean [SD], 64.44 [34.88] mm2/min vs 35.95 [18.93] mm2/min). The perforation rate was notably lower (1 out of 12 cases) than in standard endoscopy (8 out of 16). In comparison with traditional ESD, this system can establish the necessary surgical operation triangle, offering excellent flexibility, high safety, and easy, rapid assembly. However, it has several limitations. Firstly, it has to be matched with a specially-designed endoscopic system, which cannot be easily integrated with a traditional endoscope, resulting in high manufacturing costs. What is more, the length of the flexible section is limited to 65 cm, allowing access only up to the colon or the distal stomach, without the possibility for removal of dis tant lesions. Finally, the system is currently in the preclinical trial phase, with no available clinical study data.

**TABLE 1:** Comparison of different robotic systems used in endoscopic submucosal dissection

Type	Name	Year	Country	Number of operators	DOF of the robotic arm	Research progress	Surgical site	Shortcomings
One-type endoscopic robot	STRAS	2013	France	1	4	Live animal experiments (pigs)	Colorectum	Limited to the colon; lesions located ≤65 cm from the anal margin
									Flex	2013	United States	1	-	In vitro animal experiments (cattle)	Colorectum	Limited to the colon; lesions located ≤25 cm from the anal margin
									GIFTS	2022	China	1	4	Live animal experiments (pigs)	Stomach	No clinical data
	Integrated endoscopic robot	Endo MASTER	2006	Singapore	2	3	Clinical trial	Stomach, colon	Drive lag, lack of possibility to exchange instruments
										RAFE	2017	Japan	1	-	In vitro animal experiments	Stomach	No traction
										REXTER (ROSE)	2019	South Korea	2	3	Live animal experiment (pigs)	Stomach	Large size of the robotic arm
										PETH	2019	South Korea	2	4	In vitro animal experiments	Stomach	Limited multiarm space
		ETRS (EOR)	2019	Japan	1	-	In vitro animal experiments	Stomach	No clinical data
FASTER		2021	China	2	4	Live animal experiment (pigs)	Stomach, esophagus	Traction point fixation affects endoscopic operation

Abbreviations: DOF, degree of freedom; EOR, endoscopic operation robot; ETRS, endoscopic therapeutic robot system; FASTER, flexible auxiliary single-arm transluminal endoscopic robot; GIFTS, gastrointestinal flexible robotic-tool system; MASTER, master and slave transluminal endoscopic robot; PETH, portable endoscopic tool handler; RAFE, robotic-assisted flexible endoscope; ROSE, robot for surgical endoscope;

STRAS, subperichondrial transseptal system

#### The Flex robotic system 

The Flex robotic system was initially developed in the United States in 2013 as a serpentine robot system for minimally invasive cardiac surgery. It was later enhanced into the Medrobotics Flex system for head and neck surgery, particularly minimally invasive oral surgery of the oropharynx, hypopharynx, and larynx. Following the third system upgrade, a gas injection system was integrated, expanding its application to the digestive tract.^24^^;^^25^^;^^26^^;^^29^ The system received approval from the US Food and Drug Administration in 2017 for use in the digestive tract ESD. Flex comprises 4 main components: a stable platform, a console with a user interface for robot motion control, a driver for robot positioning, and a set of instrument supports. Two 4-mm forceps channels (left and right) are located on the exterior of the flexible endoscope, allowing for the insertion of dedicated forceps for manipulation. The flexible endoscope can reach lesions located up to 25 cm from the anal margin. The robotic arm has a range of nearly 180 ° and can perform grabbing, cutting, and stitching in 3D high-definition visualization. The results of a bovine cola experiment showed that the robotic system was more effective in overall lesion resection, with shorter operative times, lower perforation rates, and higher operator satisfaction scores, as compared with conventional ESD endoscopy performed by novice physicians.^30^^;^^31^ The system can be operated by a single person, with the mechanical arm extending from the specially-designed external casing, providing flexibility without being constrained by the end shell. However, certain limita tions must be considered. Flex has to be matched with a specially-designed en doscopic system, and specific rectal port for proper sealing is required to maintain inflation. The working length of the robotic endoscope is relatively short. Lesion removal is limited to 25 cm of the anal edge. Finally, current live animal and clinical study data are lacking.

#### Gastrointestinal flexible robotic-tool system

In 2022, a team of Chinese researchers led by Liang Tao introduced an ESD robotic arm with multichan- nel variable stiffness, known as the gastrointestinal flexible robotic-tool system (GIFTS). This robotic system comprises a mechanical arm and a drive system. The robotic arm features 3 pairs of rotating joints, multiple internal channels, and 3 sets of disks with variable stiffness. Each disk is 2.5-mm thick, with each group of 6 disks totaling a length of 15 mm. The outer diameter of the robotic arm is 10 mm. GIFTS is highly integrated with a 2.5 mm × 2.5 mm channel of a CCD camera, 2 3.2-mm surgical instrument channels, a 1.6-mm water-gas channel, and 2 fiber optic channels of 1.7 mm in length. The drive system consists of an external frame and internal mechanism, with 5 lines (0.3 mm) connecting the mechanical arm to the drive system: 1 for stiffness control and 4 for motion control.^32^ The maximum rotation angle of each rotating joint on the mechanical arm is set at 40 °, so the 3 pairs of rotating joints enable the mechanical arm to bend up to 120 ° up, down, left, and right, providing a total of 13 DOFs. The system was tested on diges- tive tract models and live pigs to validate the robotic arm’s performance. It also passed surgical tests on ex vivo animal tissue, demonstrating successful excision and removal of a tissue fragment. Furthermore, 1 esophageal and 4 colorec- tal ESDs were performed in 5 porcine models.^31^ The robotic arm’s outer diameter of 10 mm is comparable to the size of the single-channel gastroscope commonly used in hospitals, integrating a compact variable stiffness mechanism capable of achieving an instantaneous stiffness change of 3.55 times. In the relaxed state, the pressure exerted by the robotic arm on the digestive tract does not exceed 5 N, ensuring a stable platform for the surgical instrument and meeting the requirements of an ESD working space. However, the maximum bending angle of the robotic arm is limited due to the high integration of multiple functional channels. Furthermore, numerous randomized controlled studies are necessary to validate the application effectiveness of GIFTS, along with biocompatibility and sterilization studies to comprehensively address actual medical requirements.

### Integrated endoscopic robots

####  Endo-MASTER system 

The Endo-MASTER system is the precursor of the master and slave transluminal endoscopic robot (MASTER) system, developed by the Thant team of Nanyang Technological University in Singapore in 2006.^5^^;^^33^^;^^34^ It is a 2-arm robot with 9 DOFs in its operating arms. The tendon sheath drives 7 DOFs, while the remaining 2 DOFs (front / back translation) are controlled by direct push and pull mechanisms.^17^ The left arm features an electric hook for electrocoagulation and electrocution, and the right arm has a grasping clamp for clipping and lifting the mucosal tissue. Additionally, there is a working channel of the dual-channel endoscope for standard endoscopic instruments.^24^^;^^25^^;^^27^^;^^29^**^;^**^35^ The system requires 2 operators: an endoscopist and a surgeon. The endoscopist positions the endoscope at the bedside and controls inspiration and expiration, while the surgeon operates the robotic instruments from the central console, receiving haptic feedback. Animal studies have confirmed the feasibility and safety of Endo-MASTER for esophageal ESD, gastric ESD, and NOTES procedures, such as hepatic wedge resection and total gastrectomy.^26^^;^^33^^;^^35^^;^^36^ A total of 5 patients with early gastric neoplasia successfully underwent ESD with an average dissection time of 18.6 minutes, no perioperative complications, and a 100% R0 resection rate.^37^ The system is nearing the completion of clinical trials for superficial colorectal ESD^35^ It enhances triangulation with dexterity and tactile feedback. Drawbacks, on the other hand, include lag and nonlinear gaps due to the wire drive mechanism, inability to exchange instruments, long assembly time of about 2 hours, inability to disinfect the robotic arm, and the need for 2 operators to coordinate movements.

Robotic-assisted flexible endoscope The robotic-as-sisted flexible endoscopic (RAFE) system was developed by Tsutomu and colleagues of Kyushu University in 2017 for all commercial endoscopes. The soft endoscope enables 4 DOFs (top / bottom, left / right, front / rear, and rotation) through a handle and a turntable. The system operator secures the endoscope handle in the system bracket, operates the turntable on the endoscope handle via the internal electric wheel, and manages the air / water valve and suction valve of the endoscope through the button located under the handle.^24^^;^^26^^;^^38^** **RAFE has been subjected to 6 ESD operational tests on the isolated pig stomach. The findings indicate no significant difference in ESD operation time between RAFE and traditional endoscopy. However, the RAFE system can assist beginners in completing ESD procedures more quickly. With respect to the specified endoscope movements, the smooth motion of RAFE surpasses that of the traditional endoscopy system. Its key feature is the possibility of controlling the procedure with a single hand, streamlining the ESD operation process. Nonetheless, further clinical data are required to confirm the system applicability.

#### The REXTER robot for surgical endoscopic system 

The REXTER system is a revolute joint–based auxiliary transluminal endoscopic robot system developed by Kim and colleagues in South Korea in 2019. The system arm is mounted in series on a universal endoscope (GIF 2T240, Olympus Medical Systems, Tokyo, Japan) and can be disassembled for complex movement and operation, providing an additional range of motion. The system includes a robotic arm with 2 links (15 mm per link), actuator housing, and an interface console. REXTER is based on the tendon sheath mechanism (TSM) and provides 4 DOFs.^26^^;^^39^** **TSM is the power transmission system of the robot joint whereby the driver and a robot joint are separated. The actuator housing is equipped with a motor, a controller, and a power supply unit. When operating with REXTER, the assistant control module is required to assist in the opening and closing of the surgical device. The results of an in vitro pig model showed that REXTER can shorten ESD operation time, especially for endoscopy novices without experience in ESD.**39 **The system is characterized by simple and flexible assembly / disassembly with ordinary endoscopy according to the needs of a given surgery. It is also easy to use. However, it has certain limitations. Tissue pulling can only be performed during surgery, whereas incision, suturing, intraoperative hemostasis, and other operations cannot be performed, which precludes management of intraoperative complications.

In 2021, the team modified the prototype REXTER system to develop the robot for surgical endoscope (ROSE) system,^25^^;^^40^** **which includes a surgical manipulator, an intuitive user interface (UI), and a drive console controlling the robotic arm. The diameter of the robotic was minimized to 16 mm, which allows it to pass through a commercial supertube. The robotic arm and UI can be attached to a standard endoscope (GIF-Q260; Olympus Medical Systems). The motor, control board, and power supply unit are installed on the drive console device. The robotic arm has 3 DOFs and a clamping function. The system has completed an in vivo animal experiment^41^ involving robot-assisted resection of 16 gastric lesions in 9 live pigs. The submucosal incision speed of the expert group was significantly higher than that of the novice group (*P *= 0.002), and no complications were reported in either group. The ROSE system has several advantages. Firstly, the robotic arm can roll along the circumference of the endoscope in a front and back movement. It has good adaptability to the position of the lesion, thus minimizing the need for re-grasping. Secondly, since the structure and DOF of both the UI and mechanical arm are the same, the UI can intuitively control the arm. Also, the ROSE system can be used in conjunction with commercially available endoscopic surgical tools, such as a double electric surgical knife (Olympus). However, no comparative experiments between conventional and robot-assisted methods have yet been conducted, and the experiments that were undertaken included only the distal end of a porcine stomach, requiring further miniaturization of the system to meet the surgical needs of spatially narrow sites.

#### Portable endoscopic tool handler 

The portable endoscopic tool handler (PETH) system was designed and described in South Korea in 2019 by ^25^^;^^41^ It consists of a robotic arm, a master device, a graphic simulator, and a motor package. The arm is externally mounted, and it is capable of bending in 2 independent directions, each with a bending angle of more than 100 °, so that the tip of the instrument can reach any point within the endoscopic camera view, allowing for multidirectional traction and re-grasp-ing. The robotic arm provides an inner passage with a diameter of 2.8 mm, the same as a traditional endoscope. All parts of the robotic arm are made of stainless steel, which has been used as an effective biocompatible material for medical applications. The movement of the robotic arm is controlled by the operating master hand, intuitively bending up / down / left / right. The direction of the master hand is synchronized with the direction of the manipulator. An in vitro experiment conducted on an isolated pig stomach confirmed that PETH has obvious advantages in terms of traction, tension control, and grasping. The total operative time of PETH-ESD was significantly shorter than that of conventional ESD (*P *= 0.011), the anatomical speed was 2-fold higher (*P *<0.001), and the rate of blind dissection in ESD patients was significantly reduced (0% vs 20%; *P *<0.001). The advantage of this system is the fact that it can be integrated with a common single-channel endoscope. Also, the assembly is easy and quick, taking approximately 5 minutes. The number and direction of the robotic arms connected to the standard endoscope can be flexibly adjusted according to the type of endoscopic surgery. The transmission part of the PETH is flexible, which facilitates control of the endoscope. Having a robotic arm out of the endoscopic field of view is also helpful. However, due to the addition of a robotic arm, the PETH system increases the diameter of the endoscope to about 15 mm (the diameter of a traditional endoscope is approximately 9–13 mm), which reduces patient tolerance to endoscopic treatment. At present, only in vitro porcine stomach model study data are available for PETH, with no clinical data.

#### Endoscopic therapeutic robot system 

The endoscopic therapeutic robot system (ETRS) was developed as a master-slave robotic system by a Japanese Keiichiro team in 2019. Enhanced by the endoscopic operation robot, it can remotely control 3 endoscopic surgical instruments (grasping forceps, knife forceps, and an injection needle).^24^^;^^26^^;^^42^^;^^43^** **The left console of the system is used for operation, while the right controls the endoscopic system. The surgical device control system allows for the manipulation of each device (grasper and cutter clamp) through a handle. The latest iteration of ETRS incorporates advanced oil recovery technology featuring bidirectional tactile feedback.^29^ This system provides tactile feedback (sense of force) through the master unit, enabling the transfer of the operator’s applied force to the tip of the endoscope. This allows for all necessary operations to be performed single-handedly within the system’s range. An endoscopist seated at the console can carry out all procedural steps of ESD, including incision, dissection, submucosal local injection, water injection, air supply, aspiration, and lesion retrieval. The system has completed 7 ESD experiments on in vitro pig stomachs without any complications. It functions as a fully remote operation platform with tactile feedback, enabling remote clamp operation and auxiliary endoscopic procedures without the need of switching to another endoscope. However, issues regarding its reliability, safety, and durability persist, and further clinical data are required.

#### Flexible auxiliary single-arm transluminal endoscopic robot system 

The flexible auxiliary single-arm transluminal endoscopic robot (FASTER) system was developed by Zuo Xiuli’s team in China in 2021. The system comprises a flexible robotic arm, a drive unit, and a remote operation controller. The robotic arm primarily functions in tissue traction, connecting the tissue lift function to the tip of the universal endoscope through an elastic cap.^24^^;^^44^** **It has 4 DOFs, with a maximum angle of 90 °. The outer diameter of the additional tube is 4 mm, and the clamp diameter is 2.3 mm. The arm operates using TSM, with the robot controlled by a single rod. Movement is achieved by pushing and pulling the lever handle, transmitting motion to the robot arm to create the same 3D position based on hand-eye feedback. Two buttons are used to control opening and closing of the holder, with an emergency stop button for safety. The system requires 2 operators: an endoscopist and an assistant to control the arm remotely through a joystick and 3 buttons (open, off, and emergency stop). Previous studies analyzing 48 ESD procedures in 6 live pigs demonstrated that FASTER-assisted ESD resulted in shorter operative times, higher resection rates, and lower muscle injury and perforation rates. The system can be used for universal endoscopy without altering the original operating habits of endoscopists, and its cost is relatively low. The mechanical arm can be easily attached to or removed from the endoscope, with an assembly time of about 3 minutes. The use of a single joystick for grasping enhances the convenience of the central controller.^44^^;^^45^ However, in the coaxial arrangement of the grasping forceps and the endoscope, securing the traction point with the forceps impacts the lateral movement of the endoscope to some extent, and larger lesions may need to be grasped multiple times. Also, the feasibility and safety of the system need to be validated through numerous clinical experiments to prove its clinical applicability.

## CONCLUSIONS 

With the continuous development of science and technology, digestive endoscopic surgery robots find broad application in ESD. Compared with traditional ESD, which is complex and has a relatively limited DOF, robot-assisted ESD offers significant advantages. It can more accurately and quickly remove diseased tissue, reducing the incidence of complications. The robotic arm plays a crucial auxiliary role in ESD, enhancing procedural efficiency and safety through precise operation and high stability. Despite significant progress in digestive endoscopic surgery robots, challenges persist. These include, for example, the high cost of robotic systems, leading to their limited availability in primary hospitals. Moreover, operational complexity demands high physician skills and experience. To address these issues, it is essential to lower the costs, simplify operation processes, and enhance the intelligence of the robots. Future progress can be achieved through interdisciplinary cooperation to facilitate knowledge exchange and collaboration across different fields. Improving the design and manufacturing processes of robotic arms, along with adopting advanced control systems and algorithms, can enhance accuracy and stability while reducing costs and expanding applications.

Although technical challenges remain in ESD, continuous technological progress and innovation will lead to ongoing enhancements in digestive endoscopic surgery robots and their widespread use in ESD. These advancements will facilitate the diagnosis and treatment of early gastrointestinal cancer, contributing significantly to the promotion of human health.
